# 62859 Bringing Exposures into Mainstream Translational Research: Informatics Opportunities and Methods

**DOI:** 10.1017/cts.2021.520

**Published:** 2021-03-30

**Authors:** Ram Gouripeddi, Naomi Riches, Mollie Cummins, Katherine Sward, Julio Facelli

**Affiliations:** 1 Department of Biomedical Informatics, Center of Excellence for Exposure Health Informatics, Center for Clinical and Translational Science, University of Utah; 2 Department of Biomedical Informatics, Center of Excellence for Exposure Health Informatics, University of Utah; 3 Department of Biomedical Informatics, Center of Excellence for Exposure Health Informatics, Center for Clinical and Translational Science, College of Nursing, University of Utah

## Abstract

ABSTRACT IMPACT: This work will discuss informatics methods enabling the use of exposure health data in translational research. OBJECTIVES/GOALS: 1. Characterize gaps and formal informatics methods and approaches for enabling use of exposure health in translational research. 2. Education of informatics methods enabling use of exposure health data in translational research. METHODS/STUDY POPULATION: We performed a scoping review of selected literature from PubMed and Scopus. In addition we reviewed literature and documentation of projects using exposure health data in translation research. RESULTS/ANTICIPATED RESULTS: Primary challenges to use of exposure health data in translational research include: (1) Generation of comprehensive spatio-temporal records of exposures, (2) Integration of exposure data with other types of biomedical data, and (3) Uncertainties associated with using data as exact quantifications of exposure which are dependent on both - the proximity of measurement to subject under consideration and the capabilities of measuring devices. We identified 9 major informatics methods that enable incorporation and use of exposure health data in translational research. While there are existing and ongoing efforts in developing informatics methods for ease of incorporating exposure health in translational research, there is a need to further develop formal informatics methods and approaches. DISCUSSION/SIGNIFICANCE OF FINDINGS: Depending on the source about 50 - 75% of our health can be quantified to be a contribution of our environment and lifestyles. In this presentation, we summarize the studies and literature we identified and discuss our key findings and gaps in informatics methods and conclude by discussing how we are covering these topics in an informatics courses.

